# Interfering amino terminal peptides and functional implications for heteromeric gap junction formation

**DOI:** 10.3389/fphar.2013.00067

**Published:** 2013-05-21

**Authors:** Eric C. Beyer, Xianming Lin, Richard D. Veenstra

**Affiliations:** ^1^Department of Pediatrics, University of ChicagoChicago, IL, USA; ^2^Department of Pharmacology, State University of New York Upstate Medical UniversitySyracuse, NY, USA

**Keywords:** connexin43, connexin40, gap junction, heteromeric channel, spermine

## Abstract

Connexin43 (Cx43) is widely expressed in many different tissues of the human body. In cells of some organs, Cx43 is co-expressed with other connexins (Cx), including Cx46 and Cx50 in lens, Cx40 in atrium, Purkinje fibers, and the blood vessel wall, Cx45 in heart, and Cx37 in the ovary. Interactions with the co-expressed connexins may have profound functional implications. The abilities of Cx37, Cx45, Cx46, and Cx50 to function in heteromeric gap junction combinations with Cx43 are well documented. Different studies disagree regarding the ability of Cx43 and Cx40 to produce functional heteromeric gap junctions with each other. We review previous studies regarding the heteromeric interactions of Cx43. The possibility of negative functional interactions between the cytoplasmic pore-forming amino-terminal (NT) domains of these connexins was assessed using pentameric connexin sequence-specific NT domain [interfering NT (iNT)] peptides applied to cells expressing homomeric Cx40, Cx37, Cx45, Cx46, and Cx50 gap junctions. A Cx43 iNT peptide corresponding to amino acids 9–13 (Ac-KLLDK-NH_2_) specifically inhibited the electrical coupling of Cx40 gap junctions in a transjunctional voltage (*V*_j_)-dependent manner without affecting the function of homologous Cx37, Cx46, Cx50, and Cx45 gap junctions. A Cx40 iNT (Ac-EFLEE-OH) peptide counteracted the *V*_j_-dependent block of Cx40 gap junctions, whereas a similarly charged Cx50 iNT (Ac-EEVNE-OH) peptide did not, suggesting that these NT domain interactions are not solely based on electrostatics. These data are consistent with functional Cx43 heteromeric gap junction formation with Cx37, Cx45, Cx46, and Cx50 and suggest that Cx40 uniquely experiences functional suppressive interactions with a Cx43 NT domain sequence. These findings present unique functional implications about the heteromeric interactions between Cx43 and Cx40 that may influence cardiac conduction in atrial myocardium and the specialized conduction system.

## INTRODUCTION

Gap junctions facilitate the metabolic, biochemical, and electrical integration of component cells into functional tissues, because they contain intercellular channels that link them while excluding access to the extracellular milieu. The task of coupling the cells of the various tissues in the body is accomplished by 20 different connexin (Cx) proteins. Although the requirement for so many different connexins is not well understood and there are some functional redundancies, there are significant differences in functional properties among the channels formed of different connexins (including conductance, permeability, and gating).

Essentially all mammalian tissues (and most of the cells within them) contain more than one connexin. The expression of multiple connexins provides the opportunity for interactions with each other to form heteromeric and heterotypic channels. The properties of the resulting hetero-oligomeric channels can have a diversity of functional properties determined by their different subunits, the interactions of those subunits, and the stoichiometries of the interactions.

In previous studies, we have particularly focused on Cx43 and its interactions with other co-expressed connexins. Cx43 is one of the most widely expressed connexins. It has been found in some cells in most organs of the body, and it has been implicated in significant functions in smooth muscle, myocardium, astrocytes, lens epithelium, endothelium, etc. Many of these cells also contain other connexins. Cx43 is most commonly found with the other connexins that have the most similar sequences including Cx37, Cx40, Cx46, and Cx50 (that are members of the α sub-family of connexins encoded by the gap junction alpha (*GJA*) group of genes; Kumar and Gilula, 1992; Beyer and Berthoud, 2009).

The functional interactions of Cx43 with other connexins have been extensively studied by expression of the connexins in *Xenopus* oocytes or in transfected communication-deficient cells. Such studies have consistently shown that Cx43 will form functional heteromeric and/or heterotypic gap junction channels with three α-connexins, Cx37, Cx46, Cx50, and with Cx45 (now classified as a γ-connexin), but not with the β sub-family connexins, Cx26 and Cx32 (White et al., 1994, 1995; Elfgang et al., 1995; Brink et al., 1997; Berthoud et al., 2001; Martinez et al., 2002; Gemel et al., 2004). These functional interactions between Cx43 and other connexins may have significant functional consequences, like generation of a large variety of different channel sizes (Brink et al., 1997) or alteration of permeability, gating, and phosphorylation-dependent regulation (Elenes et al., 2001; Martinez et al., 2002). Some of these studies are supported by biochemical data showing the co-isolation of the co-expressed connexin with Cx43 in hexamers.

The ability of Cx43 and Cx40 to form functional interactions within mixed channels is controversial. Initial reports of studies conducted using *Xenopus* oocytes or HeLa cell transfectants concluded that this pair of connexins could not make functional heterotypic channels (Bruzzone et al., 1993; Elfgang et al., 1995; White et al., 1995; Haubrich et al., 1996). However, these observations were contradicted by subsequent reports of functional Cx43–Cx40 heterotypic interactions in pairs of neuro 2a (N2a) cells (Valiunas et al., 2000) and rat insulinoma (RIN) cells (Cottrell and Burt, 2001). When Veenstra and colleagues paired Cx43 with a Cx40 mutant containing substitutions of two charged residues (Musa et al., 2004), they observed symmetrically convergent alterations of voltage-dependent gating, arguing for Cx43–Cx40 heterotypic interactions (Lin et al., 2011; and unpublished results). But, in contrast, a study of connexins tagged with fluorescent proteins at their C-termini concluded that Cx40 and Cx43 only appeared to make heterotypic gap junctions when Cx45 (which could interact with either connexin) was co-expressed (Rackauskas et al., 2006).

However, the issue of heterotypic interactions between these connexins should only have importance in the rare case of a cell producing only Cx40 contacting another expressing only Cx43. In contrast, the possible heteromeric interaction of these connexins may occur frequently in cells (such as atrial myocytes and some endothelial cells) that co-express both connexins.

Several studies have supported the abilities of Cx40 and Cx43 to form functional heteromers. Mixed heteromers of these two connexins were identified by affinity purification or co-immunoprecipitation studies performed using co-expressing cells (He et al., 1999; Valiunas et al., 2001). In transfected N2A cells, Valiunas et al. (2001) observed a rather low total conductance in pairs of cells expressing both Cx40 and Cx43, with only a rather small variation in single channel conductances and alterations of voltage-dependent gating; they suggested that Cx40–Cx43 heteromers might form inefficiently and many might be non-functional. Burt and colleagues have extensively studied the consequences of Cx40 and Cx43 co-expression in A7r5 and RIN cells, including pairs of cells with different relative expression ratios (Cottrell and Burt, 2001; Cottrell et al., 2002; Burt and Steele, 2003; Heyman et al., 2009). Their data suggest that these two connexins readily form heteromeric channels and that the composition of these channels influences many properties including gating, conductance, permeability, charge selectivity, and response to platelet-derived growth factor (PDGF).

The domains within the connexin protein that influence oligomerization between subunits to form a hexamer and between different connexins to form a heteromeric hexamer have not been clearly defined. However, various biochemical and mutagenesis studies have implicated residues within the amino-terminal (NT) and within the first and third transmembrane domains (Lagree et al., 2003; Maza et al., 2005; Martinez et al., 2011). The NT has also been implicated in contributing to various channel properties including voltage-dependent gating, unitary conductance, and perm-selectivity (Oh et al., 2000, 2004; Veenstra, 2003; Musa et al., 2004; Dong et al., 2006; Gemel et al., 2006; Tong and Ebihara, 2006; Veenstra and Lin, 2006).

Some gap junction channels are very sensitive to block by polyamines like spermine and spermidine (Musa et al., 2001; Musa and Veenstra, 2003). However, this block is selective among connexins with Cx40 being very sensitive and Cx43 insensitive (Musa and Veenstra, 2003). Mutagenesis studies suggest that this connexin-specific difference is imparted by N-terminal amino acids (Musa et al., 2004; Gemel et al., 2006; Lin et al., 2006). Specifically, replacement of two negatively charged residues (E9 and E13) in Cx40 with the corresponding positively charged residues (K9 and K13) of Cx43 abolished spermine block (Musa et al., 2004). This also suggested that the block of Cx40 (but not Cx43) channels by spermine might involve interaction with these NT residues.

Polyamines (including putrescine, spermidine, and spermine) are ubiquitous polybasic molecules that interact with a wide variety of cellular molecules including nucleic acids, nucleotide triphosphates, phospholipids, and acidic proteins and influence gene transcription and translation, signaling pathways, enzyme activities, and ion channel function essential to eukaryotic cell growth and mammalian development (Igarashi and Kashiwagi, 2000; Pegg, 2009). Thus connexin channels are only one of many intracellular targets that may be modulated by cytoplasmic polyamines.

**FIGURE 1:**
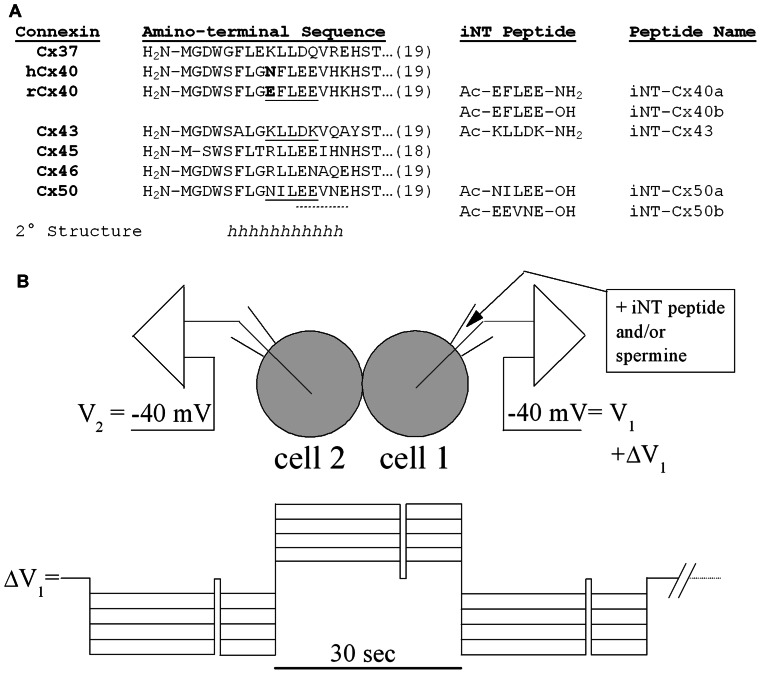
**(A)** The amino acid sequences of the first 18 or 19 amino acids are shown for six different connexins and aligned with each other. The sequences in this NT region are identical for the homologous connexins in rodents or humans except for Cx40 (which differs at position 9 as shown in bold). A substantial part of the NT domain is likely to form α-helices (h) based on consensus secondary structure predictions (Veenstra and Lin, 2006), nuclear magnetic resonance studies (Kyle et al., 2009), and homology modeling to the X-ray structure of Cx26 (Maeda et al., 2009). Synthetic pentameric NT domain peptides corresponding to underlined (continuous or dotted) connexin sequences are also shown along with their peptide names. These interfering NT (iNT) peptides were protected by amino-terminal acetylation (Ac-) and carboxy-terminal amidation (-NH2) or hydroxylation (-OH). **(B)** Diagram of the N2a cell pair configuration for the iNT peptide and/or spermine block experiments and the voltage step protocol used to apply the *V*_j_ gradients across the connexin-specific gap junctions.

The NT domain of the connexins consists of their first 22–23 amino acids. Some of the amino acids within the NT are highly conserved, while others are variable and contribute to different properties. (The NT domains of several connexins are shown in alignment in **Figure 1**.) The structure of the NT domain was initially investigated by studying synthetic peptides using circular dichroism and nuclear magnetic resonance (Purnick et al., 2000; Arita et al., 2006; Kalmatsky et al., 2009; Kyle et al., 2009). Each of these studies showed that much of the beginning of the NT is α-helical, although they differ in the exact helical region; Purnick et al. (2000) concluded that the α-helix in Cx26 extended from position 1 to 10 while Kyle et al. (2009) suggested that it extended between amino acids 5 and 15 in Cx37. When Maeda et al. (2009) determined the structure of a Cx26 channel at 3.5 Å resolution, they observed that the NT regions of the six subunits lined the pore entrance and formed a “funnel,” which restricts the diameter at the entrance of the pore. The beginning of the NT is located deep within the pore. The NT helix extends beyond the cytoplasmic side of the membrane and then forms a loop (including the highly conserved amino acids corresponding to the serine and threonine at positions 18 and 19 in **Figure 1**) that bends back to the membrane where TM1 begins. Although all of the connexins may have homologies, their NT domains do not necessarily assume identical configurations. The β-connexins were proposed to have a “glycine hinge” including and following amino acids 12 and 13 (Purnick et al., 2000; that correspond to amino acids 13 and 14 in the α-connexins); however, insertion of the SG/GG (β-connexin) motif into Cx40 or Cx43 NT abolishes *V*_j_-gating, suggesting a structural/functional disparity between the sub-families (Gemel et al., 2006).

In the current study, we examined the possibility of differential regulation of channels made of different connexins through interactions of their NT domains. Based on their inhibition by spermine, we hypothesized that Cx40 channels might be blocked by short peptides that contained similarly spaced positively charged residues, like the Cx43 sequence from residues 9–13 (KLLDK as shown in **Figure 1**). We synthesized this potentially interfering peptide (designated Cx43iNT1) and tested its ability to inhibit gap junction channel formed of various α-connexins. Because Cx37, Cx45, Cx46, and Cx50 all contain multiple glutamate residues (like Cx40 as shown in **Figure 1**), we hypothesized that they might also be susceptible to block by spermine or Cx43iNT1. In the study presented below, we tested this peptide against the different α-connexin channels and tested additional “interfering” NT (iNT) peptides based on the sequences of other connexins (**Figure 1**).

The data presented suggest that corresponding sequences of acidic and basic amino acid residues within NT domains of Cx43, Cx37, Cx40, Cx45, Cx46, and Cx50 may influence connexin-specific interactions and their abilities to function as heteromeric channels.

## MATERIALS AND METHODS

### N2a CELL CULTURES AND CONNEXIN EXPRESSION

Stable N2a cell clones expressing human Cx37, Cx40 (hCx40), or rat Cx40 (rCx40) were prepared and cultured as previously described (Veenstra et al., 1994; Lin and Veenstra, 2007; Xu et al., 2012). Mouse Cx45 (mCx45), Cx46, and Cx50 were transiently expressed in N2a cells using the pTracer^TM^ vector (Chen et al., 2011).

### CONNEXIN iNT PEPTIDE PRODUCTION

Connexin-specific NT domain peptides were synthesized in 5 mg quantities, high-performance liquid chromatography (HPLC) purified to >95%, and stored (-20°C) as lyophilized powder until needed (Anaspec, San Jose, CA, USA). The peptides were dissolved in diethylpyrocarbonate (DEPC)-treated sterile distilled water to create a stock concentration of 10 mM, stored at -20°C, and 40 μl aliquots were diluted with 140 mM KCl internal pipette solution (IPS) as needed for daily patch clamp experiments. The relevant connexin NT domain and peptide sequences are provided in **Figure 1A**. The amino and carboxyl termini of two peptides (iNT-Cx43 and iNT-Cx40b) were acetylated (amino) and amidated (carboxyl) to protect them from hydrolysis in aqueous solution. Pentameric iNT peptides for Cx43, Cx40, and Cx50 were prepared and tested in dual whole cell patch clamp experiments on homotypic Cx37, Cx40, Cx43, Cx45, Cx46, and Cx50 gap junctions expressed in N2a cells.

### GAP JUNCTION CONDUCTANCE (*g*_*j*_) MEASUREMENTS

Dual whole patch clamp experiments were performed on connexin-transfected N2a cell pairs using established procedures (Veenstra, 2001). The connexin iNT peptides were added to the patch pipette receiving the ±Δ*V*_j_ voltage clamp step for quantitative *g*_j_ measurements and calculation of the fraction of unblocked junctional current (*I*_j_) using the previously developed spermine block *V*_j_ step protocol (Musa and Veenstra, 2003; Lin and Veenstra, 2007). Equimolar spermine concentrations were added along with the iNT peptide in some experiments to assess the ability of the peptide to antagonize the *V*_j_-dependent spermine block of Cx40 gap junctions. For the peptide–spermine competition experiments, iNT peptides and spermine were added at reduced concentrations (500 μM) to conserve the amount of equimolar iNT peptide added to the patch pipette. The *V*_j_-dependent spermine block still achieved 70% inhibition of rCx40 *g*_j_, sufficient to assess the action of the iNT peptides on the block by spermine.

Patch pipettes (4–5 MΩ to patch break) were filled with a KCl IPS [in mM: KCl, 140; MgCl_2_, 1.0; CaCl_2_, 3.0; BAPTA (1,2-bis(o-aminophenoxy)ethane-N,N,N′,N′-tetraacetic acid), 5.0; HEPES (4-(2-hydroxyethyl)-1-piperazineethanesulfonic acid), 25; pH titrated to 7.4 using 1 N KOH]. The bath saline contained (in mM): NaCl, 142; KCl, 1.3; CsCl, 4; tetraethylammonium chloride (TEACl), 2; MgSO_4_, 0.8; NaH_2_PO_4_, 0.9; CaCl_2_, 1.8; dextrose, 5.5; HEPES, 10; pH 7.4 with 1 N NaOH. The osmolarity of both external and internal solutions was adjusted to 310 mOsm/L. Connexin iNT and/or spermine were added to one patch pipette at the indicated concentrations. Both N2a cells were held at -40 mV resting potential (*V*_j_ = 0 mV) and the cell (1) receiving the iNT peptides and/or spermine was stepped (Δ*V*_1_) to varying membrane potentials to produce a *V*_j_ gradient [*V*_2_ - (*V*_1_+Δ*V*_1_)]. Junctional conductance was calculated according to the equation:

$$g_{j}=-\Delta I_{2}/[\left(V_{2}-R_{el2}\cdot I_{2}\right)-\left(V_{1}-R_{el1}\cdot I_{1}\right)],$$

where Δ*I*_2_ (= -*I*_j_) is the change in whole cell 2 current (*I*_2_) during the Δ*V*_1_ step, R_el1_ and R_el2_ are the respective whole cell patch electrode resistances, and *I*_1_ and *I*_2_ correspond to the respective whole cell currents (Veenstra, 2001). To determine the fraction of *I*_j_ block induced by the iNT-Cx43 peptide or spermine, Δ*V*_1_ was alternately stepped negative, positive, and negative to the common holding potential (-40 mV) in 5 mV increments from 5 to 50 mV (**Figure 1B**). The duration of each -/+/- Δ*V*_1_ sequence was 90 s with a 500-ms step to -40 mV occurring 20 s into each 30 s -/+/- interval, as an internal *I*_j_ = 0 pA (*V*_j_ = 0 mV) baseline control measurement, followed by a 30-s rest interval. The fraction of unblocked *I*_j_ = (Δ*I*_2_(at positive *V*_j_))/(Δ*I*_2_(at negative *V*_j_)). Only those experiments where the *I*_j_ = 0 baseline remained stable throughout the total 20 min duration of the cation block protocol were used in the final analysis.

### STATISTICAL ANALYSIS

Raw data (*N* ≥ 3) from each experimental group was tested for normality by the Shapiro–Wilk test (*p*-value < 0.05) and then subjected to one-way ANOVA analysis (*f*-value < 0.05) in Origin 8.6.

## RESULTS

## Cx43 iNT PEPTIDE SELECTIVELY INHIBITS Cx40 GAP JUNCTION CHANNELS

**FIGURE 2:**
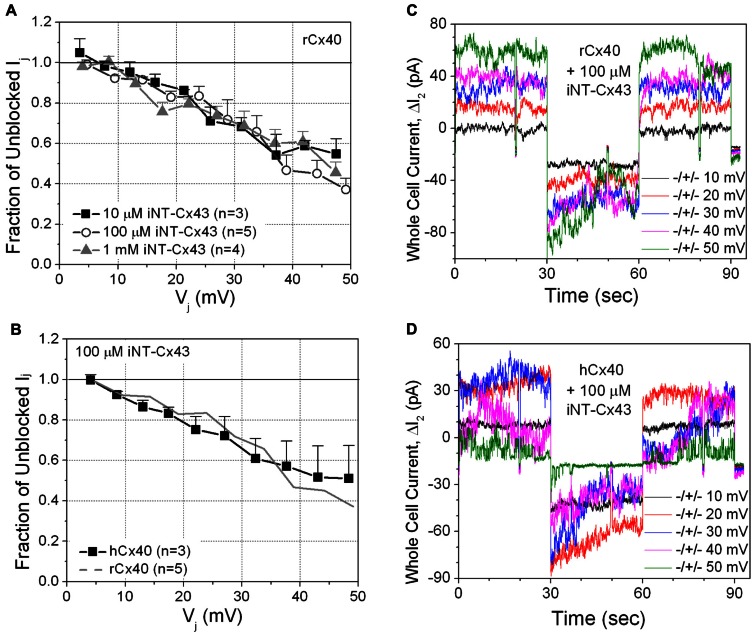
**(A)** The inhibition of rCx40 gap junction currents (*I*_j_) by unilateral addition of 10 μM (■), 100 μM (О), or 1.0 mM (∆) iNT-Cx43 peptide increased in a transjunctional voltage (*V*_j_)-dependent manner. There was no statistical difference between the three curves based on comparison of the means at each *V*_j_ value. **(B)** The *V*_j_-dependent blockade of hCx40 gap junctions (■) by 100 μM iNT-Cx43 peptide was not significantly different from rCx40 (continuous gray line). **(C)** Whole cell current traces recorded from cell 2 of a rat Cx40 cell pair with 100 μM iNT-Cx43 peptide added to cell 1 during the Δ*V*_1_ cation block voltage clamp protocol diagrammed in **Figure 1B**. The Δ*I*_2_ current (= -*I*_j_) decrease during the +*V*_1_ voltage steps (only 10 mV incremental steps shown) illustrates the block induced by the iNT-Cx43 peptide. **(D)** Actual Δ*I*_2_ current recordings from a hCx40 cell pair during an iNT-Cx43 peptide experiment illustrating a similar *V*_j_-dependent block of hCx40 gap junction currents. Gap junction channel currents are visible at ±50 mV with reduced open probability during the positive (blocking) *V*_j_ step.

The iNT-Cx43 peptide had some structural similarity to spermine: namely terminal amino groups separated by at least 10 C–C or C–N bonds. Therefore, we initially tested whether iNT-Cx43 peptide possessed any inhibitory activity toward rCx40 gap junctions. The Cx43 iNT peptide was a potent *V*_j_-dependent inhibitor of junctional conductance (*g*_j_) in cells expressing rCx40; indeed, equivalent block was observed at peptide concentrations of 10 μM, 100 μM, and 1 mM (**Figures 2A,C**).

Although Glu-9 contributes to the spermine block of rat Cx40 channels (Musa et al., 2004), this residue is replaced with an asparagine (N9) in human Cx40 (hCx40). Therefore, we hypothesized that hCx40 might show a different effect of iNT-Cx43 peptide. Surprisingly, we found that 100 μM iNT-Cx43 peptide showed a similar inhibition of both human and rat Cx40 gap junctions (**Figures 2B,D**).

**FIGURE 3:**
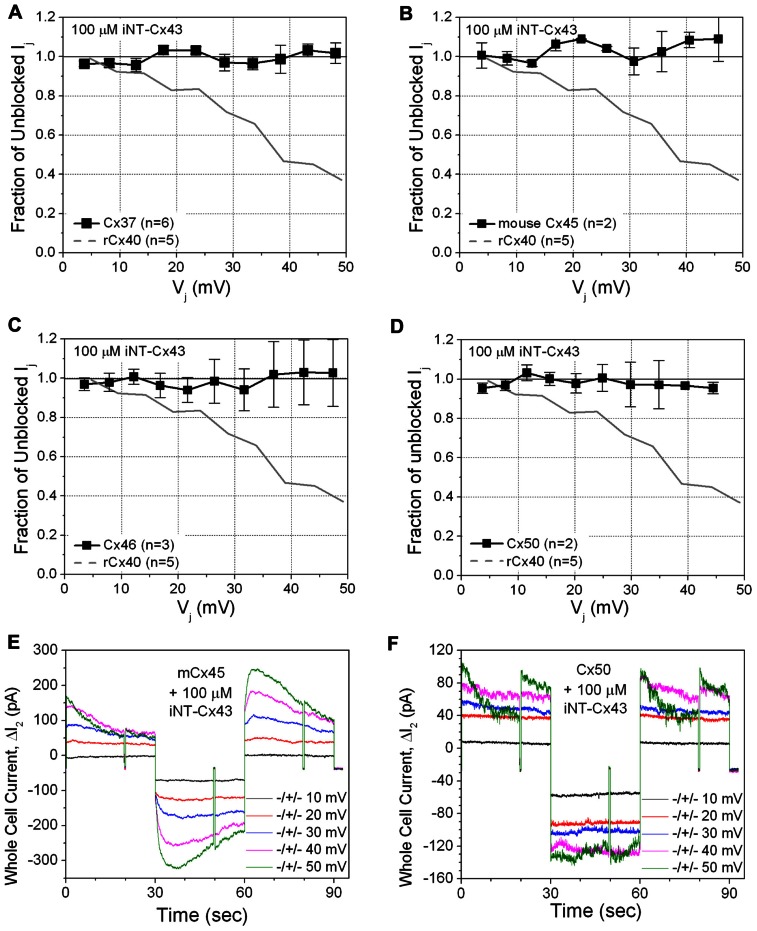
**The ability of the iNT-Cx43 peptide to inhibit Cx37 (A), Cx45 (B), Cx46 (C), and Cx50 (D) gap junctions was tested using the same *V*_j_-dependent block protocol as in Figure 2**. As a reference, the blockade of rCx40 *I*_j_ (continuous gray line) is illustrated in each panel. Unlike rCx40, none of these four connexin gap junctions were significantly inhibited by 100 μM iNT-Cx43 peptide. Actual Δ*I*_2_ current traces from Cx45 **(E)** and Cx50 **(F)** cell pair iNT-Cx43 peptide experiments illustrating the lack of *I*_j_ current block during the +ΔV_1_ (≅*V*_j_) step, in contrast to the Cx40 experiments shown in **Figure 2**. Cx45 and Cx50 gap junctions are more *V*_j_-dependent than Cx40 (half inactivation *V*_j_ = *V*_0_ or *V*_½_ ≅ ±30, ±40, or ±49 mV, respectively; Lin et al., 2006; Gonzalez et al., 2007). mCx45 gap junctions prominently display contingent hemichannel gating upon *V*_j_ polarity reversal, proposed by Harris et al. (1981), owing to the increased *V*_j_-dependence and lack of fast *V*_j_-gating kinetics of these gap junctions.

This unexpected result led us to test the ability of the Cx43 iNT peptide to interfere with the function of other connexins that also contain an ExxxE or ExxxD motif in their N-termini (**Figure 1**), Cx37, Cx46, and Cx50, or with Cx45 (which possesses only an …EE… motif). In contrast to its effects on both rat and human isoforms of Cx40, 100 μM iNT-Cx43 peptide did not affect the conductance of Cx37, Cx45, Cx46, or Cx50 gap junctions (**Figure 3**).

## Cx43iNT1 PEPTIDE EFFECTS DO NOT CORRELATE WITH SPERMINE BLOCK

**FIGURE 4:**
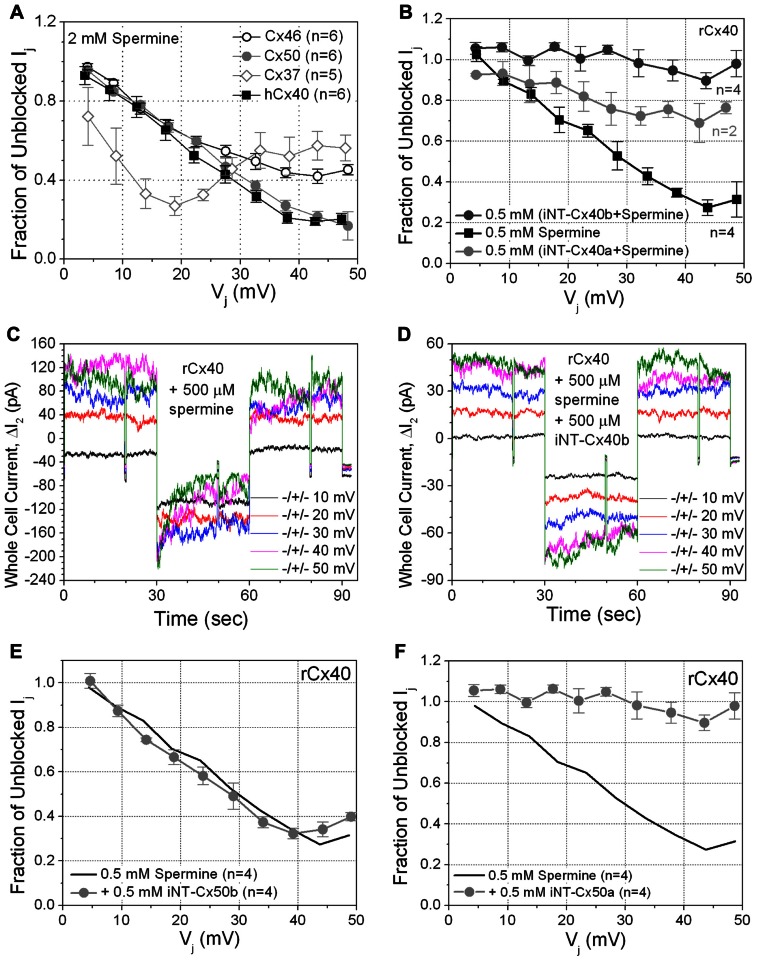
**(A)** The sensitivity of four connexin-specific gap junctions was tested using the 2 mM spermine block assay. Human Cx40 (hCx40, ■) displayed similar *V*_j_-dependent sensitivity to spermine as rCx40 despite the N9 substitution. Cx37 (♢), Cx46 (∘), and Cx50 (●) gap junctions were all ≥60% inhibited by spermine. The maximum inhibition of Cx37 *g*_j_ occurred at +20 mV, half the *V*_j_ required for maximal block of any other known connexin-specific gap junction. **(B)** The ability of iNT-Cx40 peptides to interfere with spermine block was tested by adding 500 μM spermine and iNT-Cx40a or iNT-Cx40b peptides to one patch pipette. The carboxyl- terminal hydroxylated (-OH, *z* = -4) form of the Cx40 peptide (Cx40b) effectively abolished the *V*_j_-dependent spermine block, while the amidated form (Cx40a, -NH_2_, *z* = -3) was only partially effective (ANOVA, *f*-value < 0.05). **(C)** Δ*I*_2_ current traces from an rCx40 cell pair with 500 μM spermine added to cell 1. *I*_j_ decreased during the positive 30, 40, and 50 mV *V*_j_ pulses and returned to prepulse levels during subsequent negative *V*_j_ pulses, This illustrates the time- and *V*_j_-dependent spermine block and unblock of rCx40 gap junctions. **(D)** *I*_2_ current traces from an rCx40 cell pair experiment with 500 μM spermine and the iNT-Cx40b peptide added to cell 1. Accounting for the occurrence of *V*_j_-dependent gating at *V*_j_ ≥ ±40 mV, instantaneous and steady state *I*_2_ increased in a stepwise (ohmic) fashion with increasing *V*_j_ amplitude, indicative of a lack of spermine block. **(E)** A negatively charged (*z* = -4) iNT-Cx50b peptide failed to significantly prevent the 500 μM spermine block of rCx40 gap junctions, suggesting that the bimolecular interactions between the rCx40 NT domain, spermine, and iNT peptides are not purely based on electrostatic forces. **(F)** An iNT-Cx50a peptide (based on amino acids 9–13 and possessing a carboxyl-terminal valence (*z*) of -3) significantly reduced the 500 μM spermine block of rCx40 gap junctions, suggesting a structural requirement for the interactions of iNT-Cx peptides with NT domains or spermine molecules.

Since rCx40 (but not Cx43) exhibits *V*_j_-dependent spermine block (Musa and Veenstra, 2003; Musa et al., 2004; Lin et al., 2006; Lin and Veenstra, 2007), we tested the possible relationship of iNT-Cx43 block to spermine block by examining the effects of unilateral application of 2 mM spermine on the other five connexins. Not surprisingly, hCx40 *g*_j_ was reduced in a *V*_j_-dependent manner by 2 mM spermine (**Figure 4A**, black squares).

Interestingly, spermine also caused at least some inhibition of each of the other connexins. The spermine inhibition curve for Cx50 appeared similar to that of hCx40, and the maximum inhibition of Cx50 *g*_j_ was ~80% (**Figure 4A**, gray circles). Cx46 *g*_j_ achieved 60% inhibition (**Figure 4A**, open circles). Cx37 *g*_j_ was reduced by >70% at low *V*_j_ values and then plateaued at ~50% inhibition (**Figure 4A**, gray open diamonds). Spermine inhibited Cx45 *g*_j_ by <50% (data not shown).

Thus, the selective ability of iNT-Cx43 peptide to inhibit Cx40, but not other connexin channels, does not correlate with spermine inhibition.

## iNT PEPTIDE ANTAGONISM OF SPERMINE BLOCK

Since Cx40 gap junctions were inhibited by both the Cx43iNT1 peptide and spermine, we examined whether iNT peptides based on the NT sequences of Cx40 or Cx50 (containing the ExxEE motif) could antagonize the spermine block of Cx40 gap junctions. Spermine block experiments were performed with or without the addition of iNT peptides.

The first Cx40 iNT peptide (iNT-Cx40a) was acetylated and amidated like the iNT-Cx43 peptide (**Figure 1**). iNT-Cx40a peptide was partially effective at reversing the 500 μM spermine block (**Figure 4B**, compare gray circles to spermine alone curve indicated with solid black squares). A second Cx40 iNT peptide containing the EFLEE sequence, iNT-Cx40b, 9–13 peptide was synthesized where the carboxy-terminus was hydroxylated instead of amidated to prevent the neutralization of the terminal glutamic acid group (**Figure 1**). The iNT-Cx40b peptide totally prevented the block of rCx40 by 500 μM spermine (**Figures 4B–D**, dark gray circles).

These findings suggested the hypothesis that block might result from the electrostatic interaction of the ExxEE sequence. To test this hypothesis, a hydroxylated peptide corresponding to residues 12–16 of Cx50 (EEVNE), iNT-Cx50b (**Figure 1**), was synthesized. iNT-Cx50b had no detectable effect on the block of rCx40 gap junctions by 500 μM spermine (**Figure 4E**, gray circles). However, in contrast, a second hydroxylated Cx50 iNT peptide, iNT-Cx50a, corresponding to amino acids 9–13 (NILEE; **Figure 1**) effectively eliminated the *V*_j_-dependent spermine block of rCx40 gap junctions (**Figure 4F**). This suggested a structural requirement for the abilities of these pentameric peptides to oppose the spermine block of rCx40 gap junctions.

## DISCUSSION

We began this series of experiments with a relatively simple rationale: Cx40 channels which contain the sequence ExxxE in their N-termini are inhibited by the polyamine, spermine, and might also be inhibited by a pentameric peptide derived from the NT of Cx43 which has the motif KxxxK. Indeed, we observed potent block of rCx40 channels by iNT-Cx43. The block occurred in a transjunctional voltage (*V*_j_)-dependent manner that resembled spermine block. However, the simple model of electrostatic interaction between this peptide and the connexin NT is dispelled by several other observations. The human Cx40 channel was also blocked by iNT-Cx43 despite the neutralization of the 9th residue in the human isoform (substitution of N for E). Moreover, iNT-Cx43 peptide had no effect on Cx37, Cx46, or Cx50 gap junctions, despite the presence of a similar (ExxxE or ExxxD) motif in these connexins. There also appears to be a structural requirement for the iNT effects. While a Cx50 iNT peptide containing similarly spaced negative charges had no effect, a Cx50 iNT peptide representing amino acids 9–13 antagonized spermine block of rCx40.

Our data support a selective inhibitory interaction between the NT domains of Cx40 and Cx43. iNT-Cx43 only blocked Cx40 channels. This interaction appears to have a rather high affinity, since equivalent block was achieved with 10 μM peptide to that produced by higher concentrations. This putative selective NT interaction parallels the observed functional heteromeric interactions among this group of connexins. The Cx43 iNT peptide exhibited no effect on Cx37, Cx45, Cx46, or Cx50 gap junctions, but blocked Cx40 channels. Similarly, functional heteromeric interactions between Cx43 and Cx37, Cx45, Cx46, and Cx50 have been extensively supported in the literature. We might have anticipated a reciprocal interaction of the Cx40 NT domain with Cx43 channels; however, we observed no effect when an iNT-Cx40 peptide was prepared and applied to Cx43 gap junctions (data not shown). This negative result might have been anticipated by the lack of effect of spermine on Cx43 gap junctions (Musa and Veenstra, 2003). The lack of direct reciprocity between Cx40 and Cx43 amino termini with alternately charged sequences implies that there is a structural difference between these two NT domains beyond their oppositely charged amino acids at positions 9 and 13.

Our iNT peptide data support the conclusion that Cx40 and Cx43 can form heteromeric channels, but most (if not all) of them will be non-functional (closed) due to the interactions of their amino termini. It is likely that a single Cx43 subunit is sufficient to nullify the function of a heteromeric Cx40-Cx43 hemichannel (connexon), based on the stoichiometric study of N2D and N2E mutant Cx32*Cx43E1 hemichannels showing that a single NT domain is sufficient to induce *V*_j_-dependent closure of a connexin hemichannel (Oh et al., 2000).

Finally, the connexin-specific effects of these iNT peptides also suggest the possibility of designing drugs that serve as gap junction agonists or antagonists by altering modulatory *V*_j_ or chemical gating interactions involving unique connexin domains. For instance, the Cx43 iNT peptide may antagonize the aberrant function of mutant Cx40 hemi- or gap junction channels associated with atrial fibrillation (Gollob et al., 2006; Yang et al., 2010).

## Conflict of Interest Statement

The authors declare that the research was conducted in the absence of any commercial or financial relationships that could be construed as a potential conflict of interest.
