# Erratum: Interfering amino terminal peptides and functional implications for heteromeric gap junction formation

**DOI:** 10.3389/fphar.2013.00094

**Published:** 2013-07-23

**Authors:** Eric C. Beyer, Xianming Lin, Richard D. Veenstra

**Affiliations:** ^1^Department of Pediatrics, University of ChicagoChicago, IL, USA; ^2^Department of Pharmacology, SUNY Upstate Medical UniversitySyracuse, NY, USA

There was an inadvertent error in Figure 4, panel **(F)**, pertaining to the effect of the iNT-Cx50a peptide on the spermine block of rat Cx40 gap junctions. The data that originally appeared in this figure was the same data plotted in Figure 4, panel **(B)** for the iNT-Cx40b peptide. The data in Figure 4B is correct and the correct data for the iNT-Cx50a (*n* = 4) is now illustrated in this revised version of Figure 4 for the original manuscript. The corresponding author regrets the error that occurred while configuring the figures for this manuscript and accepts sole responsibility for this mistake. The iNT-Cx50a peptide was 95% effective in preventing the inhibition of Cx40 gap junctions by 500 microM spermine and inclusion of the correct data for the iNT-Cx50a peptide does not alter the results or conclusion of the original manuscript, just the accuracy of reporting the experimental data. The legend for Figure 4 is not affected in any way and is reproduced here in its entirety.

**Figure 4:**
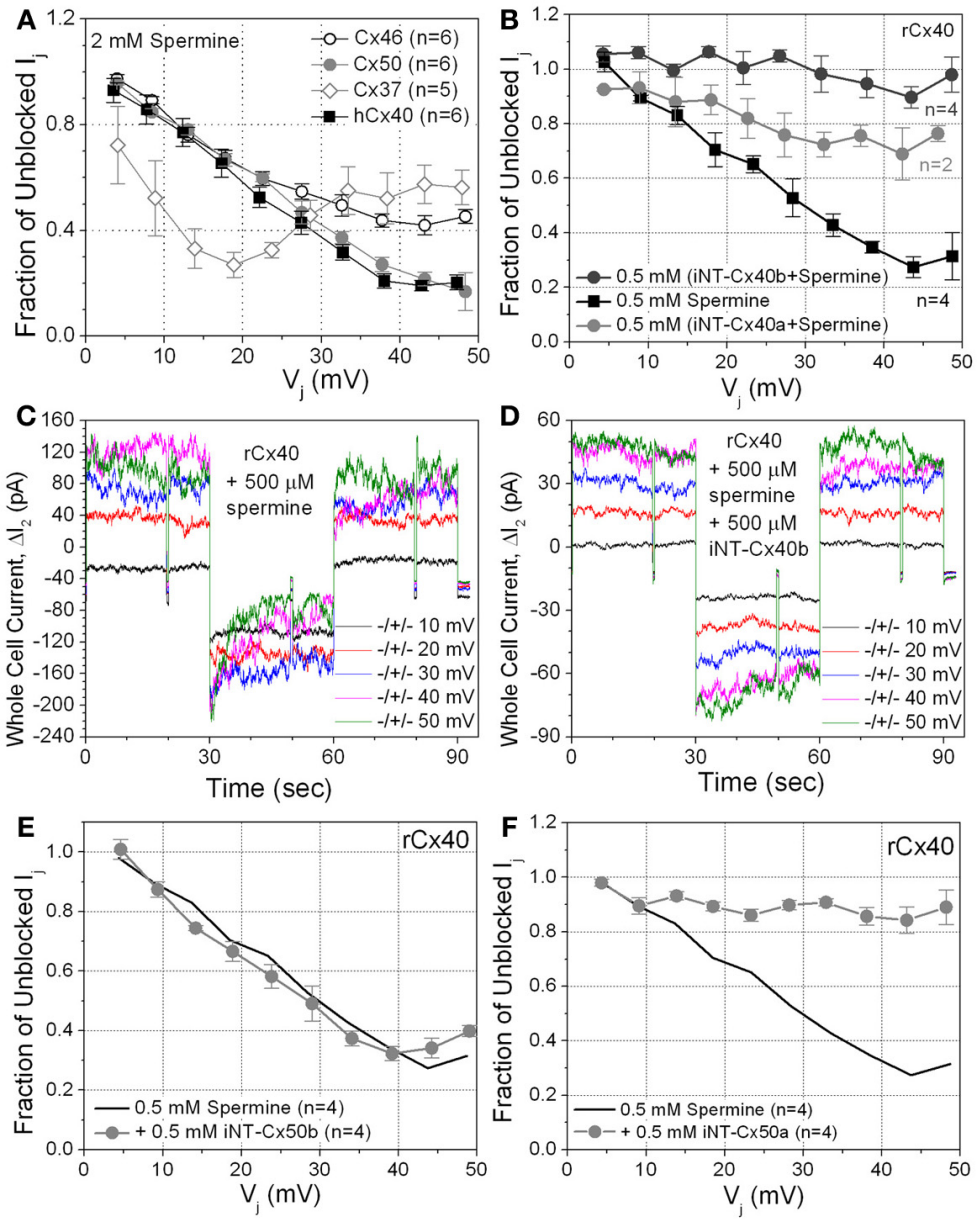
**(A)** The sensitivity of four connexin-specific gap junctions was tested using the 2 mM spermine block assay. Human Cx40 (hCx40, ■) displayed similar V_j_-dependent sensitivity to spermine as rCx40 despite the N9 substitution. Cx37 (♢), Cx46 (◦), and Cx50 (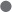) gap junctions were all ≥60% inhibited by spermine. The maximum inhibition of Cx37 g_j_ occurred at +20 mV, half the V_j_ required for maximal block of any other known connexin-specific gap junction. **(B)** The ability of iNT-Cx40 peptides to interfere with spermine block was tested by adding 500 μM spermine and iNT-Cx40a or iNT–Cx40b peptides to one patch pipette. The carboxyl-terminal hydroxylated (−OH, *z* = −4) form of the Cx40 peptide (Cx40b) effectively abolished the V_j_-dependent spermine block, while the amidated form (Cx40a, −NH_2_, *z* = −3) was only partially effective (ANOVA, *f*-value < 0.05). **(C)** ΔI_2_ current traces from an rCx40 cell pair with 500 μM spermine added to cell 1. I_j_ decreased during the positive 30, 40, and 50 mV V_j_ pulses and returned to prepulse levels during subsequent negative V_j_ pulses, This illustrates the time- and V_j_-dependent spermine block and unblock of rCx40 gap junctions. **(D)** I_2_ current traces from an rCx40 cell pair experiment with 500 μM spermine and the iNT-Cx40b peptide added to cell 1. Accounting for the occurrence of V_j_-dependent gating at V_j_ ≥ ±40 mV, instantaneous and steady state I_2_ increased in a stepwise (ohmic) fashion with increasing V_j_ amplitude, indicative of a lack of spermine block. **(E)** A negatively charged (*z* = −4) iNT-Cx50b peptide failed to significantly prevent the 500 μM spermine block of rCx40 gap junctions, suggesting that the bimolecular interactions between the rCx40 NT domain, spermine, and iNT peptides are not purely based on electrostatic forces. **(F)** An iNT-Cx50a peptide [based on amino acids 9–13 and possessing a carboxyl-terminal valence (*z*) of −3] significantly reduced the 500 μM spermine block of rCx40 gap junctions, suggesting a structural requirement for the interactions of iNT-Cx peptides with NT domains or spermine molecules.

